# Overlooked Ionic Contribution of a Chiral Dopant in Cholesteric Liquid Crystals

**DOI:** 10.3390/ma17205080

**Published:** 2024-10-18

**Authors:** Hassanein Shaban, Po-Chang Wu, Yi-Fei Jia, Wei Lee

**Affiliations:** 1 Department of Basic Science, Faculty of Engineering, The British University in Egypt, El Sherouk City 11837, Cairo, Egypt; hassanein.shaban@bue.edu.eg; 2 Institute of Imaging and Biomedical Photonics, College of Photonics, National Yang Ming Chiao Tung University, Guiren District, Tainan 711010, Taiwan; jackywu@cmmt.com.tw; 3 Unit of Product Optics Research, Cheng Mei Materials Technology Corporation, Shanhua District, Tainan 741013, Taiwan; 4 Degree Program of Photonic Technology, College of Photonics, National Yang Ming Chiao Tung University, Guiren District, Tainan 711010, Taiwan; burbeer0507@hotmail.com

**Keywords:** chiral dopant, chiral structure, ion diffusivity, electrical conductivity, impurity ions

## Abstract

This study focuses on the ionic contribution by a chiral dopant added into a nematic host for preparing cholesteric liquid crystals (CLCs). Chiral structures were designated by individually incorporating two enantiomers, R5011 and S5011, into the nematic E44 to construct right- and left-handed CLCs, respectively. Characterized by the space-charge polarization, the dielectric spectra of the CLCs were investigated in the low-frequency regime, where *f*  ≤  1 kHz. The role of the individual chiral dopant, R5011 or S5011, at concentrations of 0–4.0 wt.% in altering the ionic properties of the CLC material was analyzed by deducing the electrical conductivity, ion density, and ion diffusivity. Regardless of the cell structure to be antiparallel or twisted by 90°, a significant ionic response was observed in the right-handed CLCs in comparison with the left-handed counterparts, suggesting that excess ions originating from our R5011 were introduced into the mesogenic mixtures. This work alarms the potential contribution of notorious impurity ions by a chiral dopant, which is often ignored in fabricating CLCs for electro-optical applications.

## 1. Introduction

The electrical tuning of cholesteric liquid crystal (CLC) materials is employed in well-known display technology [1,2] and in developed optoelectronic components, such as wave plates [3,4], optical shutters [5], optical switches [6,7,8], optical diodes [9], optical modulators [10,11], and lasers [12]. Thanks to the tunable configuration and, in turn, optical state, CLCs hold great promise in the industry of smart windows as well [13]. Reflective displays based on CLCs are utilized in the flourished electronic-paper technology, to replace traditional newspapers, books, and posters, as an information media communication interface for saving the environment [14,15,16]. The most stable CLC structure has a periodically helical arrangement, which is optically characterized by Bragg’s reflection [17] in a specific range of wavelengths, which can be regarded as a photonic bandgap. The chiral structure can be practically formed by introducing a chiral agent into a nematic liquid crystal (LC) host to generate a CLC whose handedness is determined by the chirality of the dopant [18]. Such a thus-induced chiral structure in the LC texture is usually optically investigated without considering the ionic contribution by the chiral additive.

Ions or space charges inside an LC bulk are transported as a drift current induced by an externally applied electric field or as a diffusion current from a high- to low-ion-density region. Dissociated or free ions from the LC material could be produced by exposing to the ultraviolet radiation [19] or from the alignment layer due to the applied (high) voltage [20]. Water vapor or suspended fine dust particles in the air can deposit foreign charge carriers on the LC surface—in addition to the temporal ionic degradation that may occur within the LC material itself [21,22]. In the LC display industry, the ionic contamination that is regarded as a negative aspect increases the needed driving voltage and prolongs the response time; it causes technical problems such as grayscale inversion, image retention, and image flickering. As such, the incorporation of additives, especially nanoparticles such as carbon nanotubes [23,24,25,26], graphene [27], titania [28], silica [29], zirconia [30], and ferroelectric nanoparticles [31] into LCs, has been widely reported to trap mobilized ions in the presence of an external electric field.

On the other hand, the ionic effect, apparently undesired as mentioned above, can actually serve to improve the electro-optical properties of CLCs on some occasions by provoking new optical textures on the basis of the electrohydrodynamic effect. The threshold frequencies and voltages to induce these new states have been characterized [32,33]. A chiral ionic liquid was utilized in establishing switching between various optical textures and in broadening the CLC reflection band [34,35]. The ion trapping inside the polymer network of a polymer-stabilized CLC was demonstrated to expand the width of the reflection band under a direct-current (DC) electric field [36,37]. Negative CLC material (possessing negative dielectric anisotropy) was employed in developing electrically controlled lasers founded on the electrohydrodynamic effect [38].

Despite intensive efforts in developing CLC mixtures for electro-optical applications by involving foreign ionic additives, the ionic contribution by a chiral dopant to electrical properties of the CLC has been scarcely explored. Undoubtedly, impurity ions originating from a chiral dopant in a CLC material could influence the ionic properties and the response to the applied electric field. Often being disregarded, the potential ionic effect of an ion-rich chiral dopant should not go unnoticed in the fabrication of a CLC.

In this work, the ionic properties of several CLC samples were investigated. These CLCs were prepared by the nematic host E44 doped individually with right-handed R5011 and left-handed S5011 at concentrations of 0.5–4.0 wt.%. Corresponding to the chiral dopant’s concentration, the phase transition temperature from the isotropic to the CLC phase was determined by the temperature-dependent real part of the dielectric spectrum and the deduced first derivative with respect to the temperature. Examination was undertaken for the low-frequency imaginary part of the dielectric function to grasp ionic contribution in the pure E44 sample or doped E44 containing either R5011 or S5011. The thermal behavior of the dielectric-loss parameter was studied over a temperature range of 60° (i.e., 20–80 °C), and dielectric parameters were derived to enable analysis of the ionic behavior. The CLCs were inspected in both the antiparallel and 90°-twisted cell modes for exploring variations in electrical and optical measurements. Optical transmission emerging from right- and left-handed CLC structures was explored in a wider temperature range of 20–90 °C. Optical features for characterizing transmission were derived to describe the thermo-optic response. A binarily doped CLC was also established by mixing equal concentrations of R5011 and S5011 to form a racemic structure for the study of the ionic contribution varying with temperature. This study serves as an alert, highlighting the potential impact of impurity ions from chiral dopants, which is often overlooked in the fabrication of CLCs for electro-optical applications.

## 2. Materials and Methods

### 2.1. Materials

**Figure 1:**
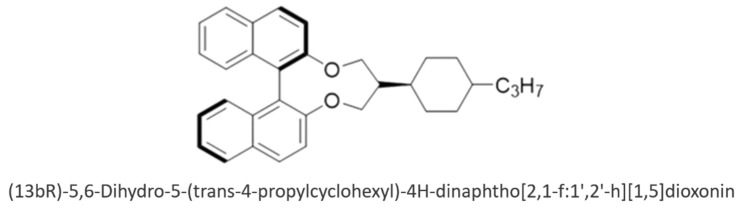
Scheme of the molecular structure of the chiral dopant R5011.

Various CLCs were prepared by mixing a eutectic nematic LC mixture; E44 was composed of plural cyano-mesogenic compounds (Daily Polymer Industry Co., Ltd., Kaohsiung, Taiwan), with a right-handed chiral dopant, R5011 (CAS No. 944537-61-5), or its left-handed counterpart, S5011 (CAS No. 693227-30-4), (Jiangsu Hecheng Display Technology, Co., Ltd., Nanjing, China) at concentrations of 0.5–4.0 wt.%. The helical twisting power (HTP) of R5011 and S5011 in E44 is 107.5 μm^−1^. According to the datasheet provided by the supplier, the purity grade of the two batches is 98% by gas chromatography. Figure 1 shows the molecular structure of the right-handed enantiomer, whose chemical formula is C_32_H_34_O_2_ and molecular weight 452.62704 Da.

### 2.2. Preparation of LC Cell Samples

The nematic LC host E44, with extraordinary refractive index *n*_e_ = 1.7904 and ordinary refractive index *n*_o_ = 1.5484 at wavelength *λ* = 589 nm and dielectric constants *ε_‖_* = 22.4 and *ε*_⊥_ = 5.2 (giving positive dielectric anisotropy Δ*ε* = 17.2) at frequency *f* = 1 kHz and temperature *T* = 20 °C, was used as the host material for the added chiral dopants in this study. The weight concentration of either enantiomer, S5011 or R5011, in E44 was precisely determined via a micro-balance (Mettler Toledo X26 Delta Range) with extremely high weighing precision (10^−6^ g). Three LC/chiral dopant material compositions were prepared, including singly doped mixtures, E44/R5011 and E44/S5011, as well as a binary-doped E44/R5011/S5011 mixture. In order to ascertain the uniformity of a mixture, the prepared solution was placed on a magnetically stirring heating platform (IKA C-MAG) and heated up to the isotropic phase temperature and slowly stirred for one hour. The binarily doped sample, E44/R5011/S5011, was concocted with E44/R5011 and E44/S5011 at equal dopant concentrations at a ratio of 1:1. Therefore, E44/R5011/S5011 with 1.0 wt.% binary dopant contains 0.5 wt.% R5011 and 0.5 wt.% S5011 in E44. Each commercial LC cell (Mesostate Co., Ltd., New Taipei, Taiwan) is composed of two transparent glass substrates coated with indium–tin oxide (ITO) to provide a conductive electrode area, *A* = 1 cm^2^. The electrode surface was covered with a planar-aligning polyimide film, SE-2170. Two types of cell configurations were exploited: the conventional planar structure (in an antiparallel or 180° planar cell) and the 90°-twisted mode (enabled by a 90°-twisted nematic cell), with cell gap dimensions of 6 μm and 5 μm, respectively. According to the relationship between the cell gap *d* and the capacitance *C*, *d* of an empty commercial cell was confirmed by measuring *C* of the enclosed air layer using an LCR meter (HIOKI 3522-50 LCR HiTester, Ueda, Nagano, Japan). Pure and doped E44 were injected into empty cells at *T* = 110 °C by capillary action to avoid aligning LC molecules along the filling direction. Finally, the open edges of a cell were sealed with AB glue, thereby protecting the confined LC from environmental contaminations.

### 2.3. Phase Transition Temperature Measurement by Dielectric Spectroscopy of LCs

As a fast, simple and highly accurate method, temperature-dependent dielectric spectroscopy was employed to investigate the phase transition behavior and phase transition temperature of the LC materials [39]. The LC–isotropic phase transitions of E44 without and with incorporated chiral dopants were studied by dielectric measurement utilizing a precision LCR meter (Agilent E4980A, Santa Rosa, CA, USA) at a cooling rate of 1 °C/min governed by a temperature controller (Linkam T95-PE, Salfords, Surrey, UK) over the temperature span of 20–110 °C. The frequency range of the measured real-part dielectric spectrum is 20–2 × 10^6^ Hz, determined by probe voltage of 0.3 V_rms_. The phase transition sequence of a LC material can be observed from the *T*-dependent real part of dielectric function, *ε*′ (*T*), and further specified by its first-order differentiation with respect to *T*, d*ε*′/d*T* at a fixed *f* (say, 10 kHz, as long as *ε*′ is locally independent of *f*).

### 2.4. Thermo-Optical Transmission Measurement of CLCs

The Bragg reflection band generated from the periodically helical structure was monitored in the visible spectrum (*λ* = 400–800 nm) via a fiber-optic spectrometer (Ocean Optics HR2000+, NIR512, Orlando, FL, USA), together with a halogen lamp (Ocean Optics HL-2000-CAL, Orlando, FL, USA). Transmission spectra of the CLC samples in both the planar and 90°-twisted cell modes were taken in the temperature range of 20–90 °C regulated by a thermoelectric cooling system (TEC Controller CDS15008RRA).

### 2.5. Measurement of Ionic Properties of CLCs

Rooted in the advantages of dielectric spectroscopy, known as a rapid and nondestructive measurement with a wide frequency range, the behavior of ions within an LC can be thoroughly analyzed [21]. For this study, perpendicular-component dielectric spectra in the range of *f* = 1 mHz–100 kHz were acquired with an LCR meter (HIOKI 3522-50 LCR HiTester) linked with a GPIB interface for computer control by the graphical-user-interfaced software LabVIEW 8.6. The probe voltage for dielectric measurement was 0.3 V_rms_, which was lower than the threshold voltage of E44 (~0.9 V_rms_) to avoid causing reorientation of LC molecules [40]. The dielectric properties of the examined materials were monitored over a temperature range of 70 °C (from 20 to 90 °C). For a stable measurement, each dielectric spectrum was measured after waiting for 10 min until thermal equilibrium was attained between the cell substrates and the CLC material sandwiched in the cell.

**Figure 2:**
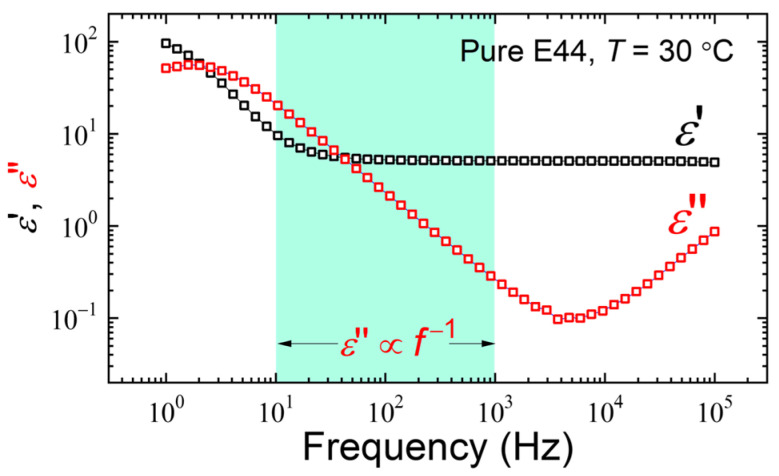
Perpendicular component of complex dielectric spectrum of an antiparallel cell of E44 in the frequency range of 1 Hz–100 kHz at 30 °C.

Figure 2 shows the real-part (*ε*′ (*f*)) and imaginary-part (*ε*″ (*f*)) dielectric spectra of the nematic LC E44 at 30 °C. Note that the *ε*′ value at *f* > 1 kHz is frequency-independent, whereas at lower frequencies (say, *f* < 10 Hz) the space-charge polarization of contained ions within the LC bulk in response to the applied alternating-current (AC) signal becomes significant, bringing about higher measured *ε*′. Because of the contribution of the ions to the polarization, *ε*′ varies with *f*^−3/2^ in the dispersion range and *ε*″ changes with *f*^−1^ over the frequency regime of 10 Hz < *f* < 1 kHz (as designated by the shaded region in Figure 2), according to the following equations [28]:

(1) $$\varepsilon^{\prime }=\frac{nq^{2}D^{3/2}}{\pi^{3/2}\varepsilon_{0}dk_{B}T}f^{-3/2}+{\varepsilon^{\prime }}_{b}$$

and

(2) $$\varepsilon^{\prime \prime }=\frac{nq^{2}D}{\pi \varepsilon_{0}k_{B}T}f^{-1},$$

where *ε*_0_ (≈8.854 × 10^−12^ F·m^−1^) is the electric permittivity in vacuum, *k*_B_ (=1.380649 × 10^−23^ J·K^−1^) is the Boltzmann constant, *q* is the electric charge, *n* is the ion density, *D* is the diffusivity, *d* is the cell gap, *ε*′_b_ is the intrinsic dielectric constant of the LC bulk material, and *T* is the absolute temperature in Kelvin. It is worth reminding that Figure 2 shows measured data, and the graphical behaviors are log–log nonlinear, whereas the behaviors of Equations (1) and (2) stem from a theoretical model, which is valid in a more limited range and exhibits log–log linearity.

## 3. Results and Discussion

### 3.1. Influence of the Chiral Dopant Concentration on CLC Phase Transition Temperature

**Figure 3:**
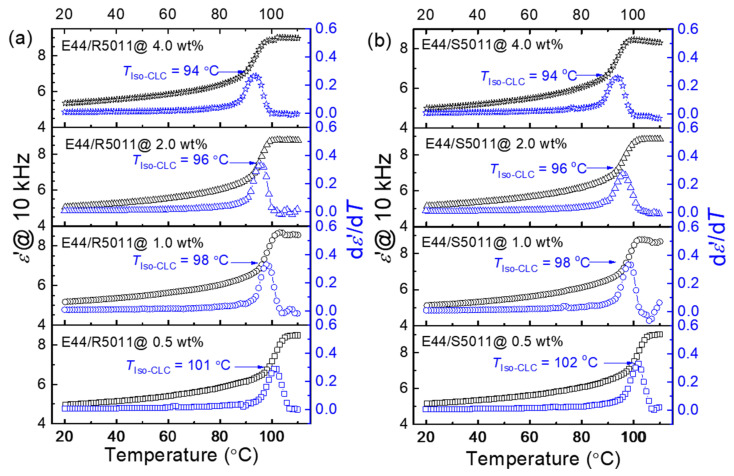
Determination of the phase transition temperatures of E44 doped with (**a**) R5011 and (**b**) S5011 at various concentrations of 0.5, 1.0, 2.0, and 4.0 wt% (in antiparallel cells) by measuring *T*-varying *ε*′ (black symbols) and calculating the first derivative d*ε*′/d*T* (blue symbols) at a fixed frequency of 10 kHz.

*T*-dependent *ε*′ was observed at 10 kHz, and its first derivative (d*ε*′/d*T*) was deduced over 20–110 °C at a cooling rate of 1 °C/min. The phase transition causes a fluctuation in the thermal behavior of *ε*′, which can be monitored by finding the corresponding temperature to the peak of the *T*-varying d*ε*′/d*T* curve. Figure 3a shows both the *ε*′ and d*ε*′/d*T* curves of E44 doped with 0.5, 1.0, 2.0, and 4.0 wt.% of R5011 (having right-handed chirality) at 10 kHz. For each loading of R5011, the highest *ε*′ value occurred approximately at 110 °C within the isotropic phase. When the temperature was lower than the phase transition temperature (precisely, the clearing point *T*_c_), *ε*′ began to fall with decreasing *T*. The measured *ε*′ value can be represented as the effective dielectric constant

(3) $${\varepsilon^{\prime }}_{\mathrm{eff}}={\varepsilon^{\prime }}_{∥}\sin^{2}\theta +{\varepsilon^{\prime }}_{⊥}\cos^{2}\theta ,$$

where *θ* is the tilt angle of the LC principal axis (also known as the director) measured from the substrate plane, and *ε*′*_‖_* and *ε*′_⊥_ (<*ε*′*_‖_* for E44) are the parallel and perpendicular components of the dielectric constant, respectively. By lowering *T*, the LC molecules became more ordered with a collective alignment, leading to a gradual decrease in *θ* to reflect the effect of the planar anchoring on the LC easy axis. In accordance with Equation (3), the decrease in *ε*′_eff_ was a result of the reduced thermal fluctuation or diminishing tilt angle, reaching to minimal *ε*′_eff_ (≈*ε*′_⊥_) when the field lines passed vertically through the planar-aligned LC structure at 20 °C. The increase in R5011 and S5011 concentrations from 0.5 to 4.0 wt% procured a drop in transition temperature *T*_Iso-CLC_ (i.e., *T*_c_) from 101 to 94 °C (Figure 3a) and 102 to 94 °C (Figure 3b), respectively. The results indicate that the effects of R5011 and S5011 on the phase transition temperature are similar, as would be expected regardless of the handedness of the induced CLC structures of E44/R5011 and E44/S5011. Moreover, the gained chirality due to packing between the nematic LC molecules and chiral molecules can slightly weaken the collective intermolecular interactions, which lowers the latent heat required to transform CLC into the isotropic phase.

### 3.2. Thermal Behavior of Ionic Responses of Oppositely Handed CLCs

**Figure 4:**
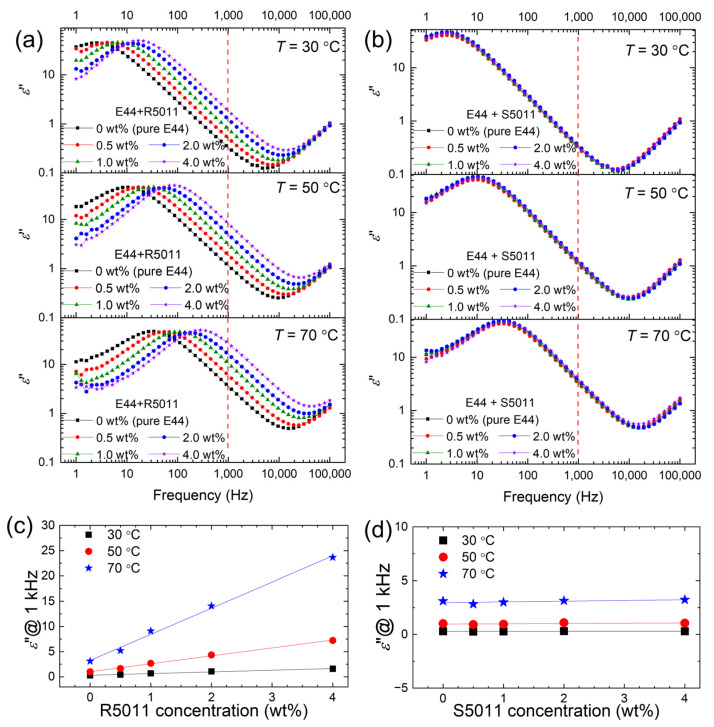
*ε*″ spectra in the frequency range between 1 Hz and 100 kHz of E44 doped with (**a**) R5011 and (**b**) S5011 at concentrations of 0, 0.5, 1.0, 2.0, and 4.0 wt.% in antiparallel cells at 30, 50, and 70 °C, and the measured *ε*″ at 1 kHz (extracted from the data labeled with the dashed red lines in (**a**,**b**) varying with the concentration of the chiral dopants (**c**) R5011 and (**d**) S5011 at the three temperatures, where the solid lines are the linear fitting curves of the experimental data.

Figure 4 shows the *ε*″ spectra measured at 30, 50, and 70 °C for both E44/R5011 and E44/S5011 CLCs containing various concentrations—0.5, 1.0, 2.0, and 4.0 wt.%—of the designated chiral dopant. Here, a pure E44 cell as a reference was included in the measurement. A strong dependence of *ε*″ on *f* was noted in the frequency range *f* > 10 kHz, which is dominated by a pseudo-dielectric relaxation phenomenon called the ITO effect in LC cells rather than an intrinsic relaxation behavior of the LC molecules [41]. In the range of 10 Hz < *f* < 10 kHz, *ε*″ varied with *f*^−1^, as expressed by Equation (2). The dielectric loss in this frequency range is mainly contributed by the free space charges, and a higher *ε*″ value representing a greater loss in the applied electric energy specifies a higher conductivity.

Comparing the spectra of E44/R5011 with those of pure E44, one can see from Figure 4a that the space-charge polarization blueshifted (to a higher frequency) as the concentration of R5011 increased. Extracted from Figure 4a, Figure 4c discloses that *ε*″ (at *f* = 1 kHz) was linearly dependent on the dopant concentration, implying the essential contribution of free ions emanating from the chiral additive. On other hand, the *ε*″ spectra of E44 doped with S5011 at various concentrations were almost superimposed on the spectrum of pure E44 at a given temperature (Figure 4b). Figure 4d clearly shows that *T*-dependent *ε*″ (at 1 kHz) was virtually unrelated to the S5011 concentration, confirming no appreciable input of free ions from S5011. Notably, the frequency range of the inversely linear region (*ε*″ ∝ *f*^−1^), which signifies the space-charge polarization in the *ε*″ spectrum of E44, moved from 10 Hz–10 kHz to 200 Hz–50 kHz as *T* rose from 30 to 70 °C (Figure 4a,b). This *T*-dependent blueshift, observed in both types of CLC samples, aligns with the typical thermal response of contained ions. The increase in ion concentration in E44/R5011 evoked a more pronounced thermal response in *ε*″, as shown in Figure 4c, whereas samples doped with S5011 did not exhibit further variations, and the thermal change in *ε*″ was completely caused by the native ions comprised in the E44 host (Figure 4d). The thermal response of the *ε*″ spectrum was remarkable despite the dopant concentration of either R5011 or S5011. The dielectric relaxation signal in the *ε*″ spectrum shifted to a higher frequency with elevated temperature even in the sample of 0 wt.% chiral guest, referring to the response from impurity ions existing in pure E44.

**Figure 5:**
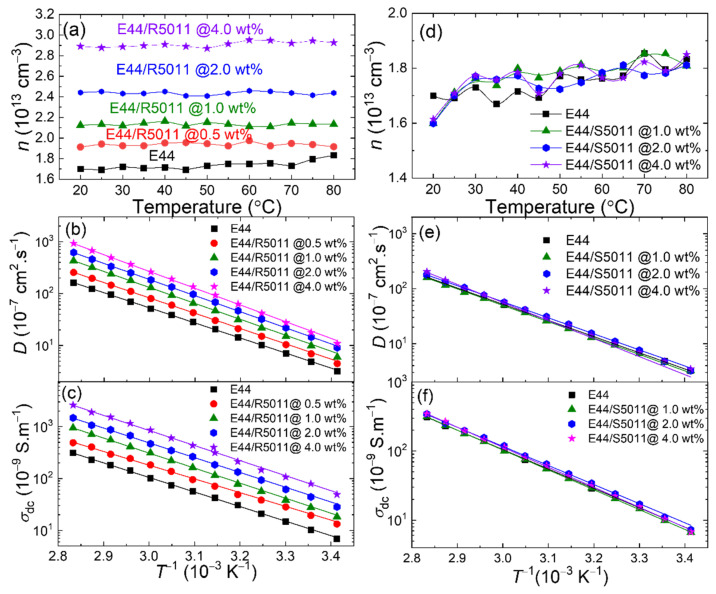
Thermal study of (**a**) the ion density *n,* (**b**) diffusivity *D*, and (**c**) electrical conductivity *σ*_dc_ in E44/R5011 and of (**d**) the ion density *n*, (**e**) diffusivity *D*, and (**f**) electrical conductivity *σ*_dc_ in E44/S5011 containing various chiral-dopant concentrations between 0 and 4.0 wt.% in antiparallel cells through a temperature range spanning from 20 to 80 °C. Solid lines represent linear fitting of the natural log of *D* in (**b**,**e**) and of *σ*_dc_ in (**c**,**f**) against the inverse absolute temperature (in K^−1^) in compliance with the Arrhenius equation.

To further investigate the ionic behaviors of E44 incorporated separately with R5011 and S5011, and the real and imaginary parts of the dielectric spectra were fitted with Equations (1) and (2) to obtain the ion density *n* of mobile space charges and diffusivity *D*. AC conductivity *σ*_ac_ of the LC material is a vital parameter usually taken into serious consideration in the LCD industry. It can be derived from the measured *ε*″ spectrum by the relationship *σ*_ac_ (*f*) = 2*πfε*_0_*ε*″ (*f*). DC conductivity *σ*_dc_ can then be acquired from Jonscher’s universal power law,

(4) $$\sigma_{\mathrm{dc}}=\sigma_{\mathrm{ac}}(f)-sf^{m},$$

where *s* and *m* are the fitting parameters. The ion density *n*, in the case of E44/R5011, increased from 1.7 × 10^13^ cm^−3^ at a zero dopant concentration to 2.9 × 10^13^ cm^−3^ for the sample dispersed with 4.0 wt.% R5011. The increase by 70% suggested the release or dissociation of free ions from the dopant (i.e., R5011) molecules (Figure 5a). Whereas in the case of E44/S5011, the *n* values fluctuated around 1.6–1.9 × 10^13^ cm^−3^ and were somewhat similar to those of the pure E44 reference, meaning that the ions contributed by S5011 doped in E44 were limited (Figure 5d). Furthermore, the ion density showed a seemingly thermal stability over the temperature range of 20–80 °C, suggesting that the content of mobile ions was independent (Figure 5a) of or weakly dependent (Figure 5d) on the temperature in E44/R5011 or E44/S5011 samples.

Figure 5b,e reveal the change in diffusivity of free ions with varying temperature for right-handed (E44/R5011) and left-handed (E44/S5011) CLC samples, respectively. Here, the temperature scale is represented in the reciprocal of the Kelvin scale from 3.41 × 10^−3^ to 2.83 × 10^−3^ K^−1^, corresponding to the Celsius temperature scale from 20 to 80 °C. The noted increase in *D* of free ions in the logarithmic scale with rising *T* in a CLC can be linearly described by the Arrhenius model, implying the increase in gained kinetic energy from the applied thermal energy. The application of thermal energy gave rise to enhanced diffusion of ions driven by an AC electric field, as indicated by the shift of *ε”* to a higher frequency, as shown in Figure 4. Figure 5d illustrates how the increase in R5011 concentration promoted the *D* value. For example, *D* = 12.3 × 10^−7^ cm^2^·s^−1^ in E44 was doped with 4.0-wt.% R5011 at 20 °C. It was raised to 920.9 × 10^−7^ cm^2^·s^−1^ at 80 °C. In contrast, the thermal behavior of *D* was similar over all S5011 concentrations from 0 to 4.0 wt.% in the E44/S5011 samples (Figure 5e). The *D* values of the E44/S5011 samples were all around 3.2 × 10^−7^ cm^2^·s^−1^ at 20 °C, which increased to 162.3 × 10^−7^ cm^2^·s^−1^ at 80 °C.

Figure 5c,f show the change in DC conductivity with *T* for the E44/R5011 and E44/S5011 samples, respectively. At each chiral dopant concentration, the *σ*_dc_ value of an LC cell increased with increasing *T*, and this trend can be again fitted to the Arrhenius formula. As depicted in Figure 5c, when the concentration of R5011 increased from 0 to 4.0 wt.% at *T* = 20 °C or 1/*T* = 3.41 × 10^−3^ K^−1^, the DC conductivity *σ*_dc_ (*T*) grew from 6.88 × 10^−9^ to 39.8 × 10^−9^ S·m^−1^, which conforms with the dependence of *σ*_dc_ on the ion density *n* and the ion mobility *µ* described by

(5) $$\sigma_{\mathrm{dc}}=nq\mu$$

At a fixed temperature, the DC conductivity in E44/S5011 samples (with doping concentrations of 0–4.0 wt%) spanned narrowly between 6.45 × 10^−9^ and 7.24 × 10^−9^ S·m^−1^, which was close to that of pure E44 and 6.88 × 10^−9^ S·m^−1^. The maximal difference of 0.79 × 10^−9^ S·m^−1^ can be regarded as systematic uncertainty arising from the dielectric and the temperature measurements (Figure 5f).

### 3.3. Thermal Behavior of Ionic Responses of LCs with Binary Doping of Two Racemic Chiral Dopants

**Figure 6:**
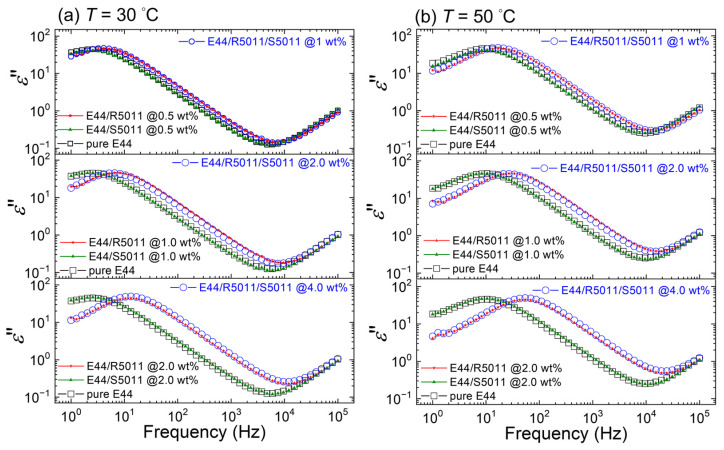
*ε*″ spectra of pristine E44; E44 doped separately with 0.5, 1.0, and 2.0 wt% concentrations of R5011 and S5011; and E44 doped with 1.0, 2.0, and 4.0 wt% of R5011/S5011 as a binary dopant at (**a**) 30 °C and (**b**) 50 °C. The samples are confined in antiparallel cells of 6 μm in thickness.

To inspect the influence of chiral dopants on the ionic properties of a racemic structure, E44/R5011 and E44/S5011 samples of equal dopant concentrations were mixed in a weight ratio of 1:1 to form achiral E44/R5011/S5011 samples. Figure 6a,b delineate the measured *ε*″ spectra of the samples with 1.0, 2.0, and 4.0 wt.% R5011/S5011 concentrations at 30 and 50 °C, respectively. For comparison, included as well are the measured *ε*″ spectra of pure E44 and doped E44 comprising individually the enantiomers R5011 and S5011 at concentrations 0.5, 1.0, and 2.0 wt%. In the frequency range characterized by space-charge polarization, where *ε*″ varies with *f*^−1^, the *ε*″ spectra of the E44/R5011/S5011 samples exhibited a noticeable blueshift with increasing R5011/S5011 concentrations from 1.0 to 4.0 wt.%. This blueshift was also found in E44/R5011 spectra with increasing R5011 contents from 0.5 to 2.0 wt.%. Notably, the *ε*″ spectra of E44/R5011/S5011 and E44/R5011 significantly overlapped as a result of the major ionic contribution by R5011 as discussed in Section 3.2 (Figure 5). On other hand, the *ε*″ spectra of the E44/S5011 sample did not show an observable response to the added S5011 at concentrations between 0.5 and 2.0 wt.%, and they superimposed the pure E44 spectra, inferring the neglectable ion contribution by S5011 in the singly doped E44/S5011 and binarily doped E44/R5011/S5011 samples. The same experiment was repeated at 50 °C. The measured *ε*″ spectra of E44/R5011, E44/S5011, and E44/R5011/S5011 samples all showed blueshifts in the frequency regime with space-charge polarization in dominance, connoting that the enclosed ions gained substantial energy at a higher ambient temperature.

**Figure 7:**
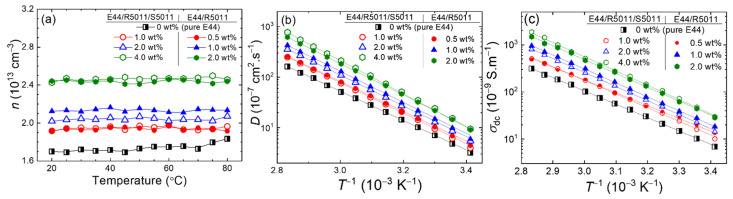
Thermal study of (**a**) the ion density*,* (**b**) ion diffusivity, and (**c**) DC conductivity deduced from the *ε*″ spectra of pure E44; E44 doped individually with 0.5, 1.0, and 2.0 wt.% of R5011; and E44 binarily doped with 1.0, 2.0, and 4.0 wt.% of R5011/S5011 at temperatures 20–80 °C. Solid lines represent linear fitting of logarithmic *D* in (**b**) and of *σ*_dc_ in (**c**) against the inverse absolute temperature (in K^−1^) following the Arrhenius equation. All the LC samples are in the antiparallel cell mode.

Figure 7a reports ion densities in pure E44, E44/R5011, and E44/R5011/S5011 samples at distinct doping concentrations over a temperature range of 20–80 °C. The ion densities of the E44/R5011 and E44/R5011/S5011 samples were higher than those of E44 and increased with increasing chiral-dopant concentration. In particular, we found that the ion densities in the 1.0, 2.0, and 4.0 wt.% E44/R5011/S5011 samples were close to those of the E44/R5011 samples containing 0.5, 1.0, and 2.0 wt.% R5011, respectively. The ion density did not display a clear thermal dependence; it fluctuated with a small difference in each examined sample over the temperature range of 60 °C. Additionally, Figure 7b depicts the increase in diffusivity for E44/R5011 and E44/R5011/S5011 samples with increasing chiral-dopant content at each *T* value. The diffusivities of the E44/R5011/S5011 samples consisting of 1.0, 2.0, and 4.0 wt.% of the binary dopant were almost the same as those of the E44/R5011 samples comprising 0.5-, 1.0-, and 2.0-wt% R5011. It was confirmed by comparing with E44 that the increases in ion density and diffusivity in the E44/R5011/S5011 samples were mainly caused by R5011 (as one of the additive components) of the binary dopant. Moreover, the thermal behavior of the diffusivity satisfies the Arrhenius equation as the following:

(6) $$D(T)=D_{0}\exp\;\left(-\frac{E_{a}}{k_{B}T}\right),$$

where *D*_0_ is the diffusivity at 0 K, and *k*_B_ denotes the Boltzmann constant as aforementioned in Section 2.5, which is equivalently ca. 8.617 × 10^−5^ eV·K^−1^. Linear regression was performed to determine the activation energy *E*_a_ of mobile ions in the LC phase. Their values are summarized in Table 1.

Activation energy signifies the energy needed to overcome the barrier when the material state changes or a chemical reaction occurs [28,42]. It was found that the *E*_a_ of ions in pure E44 was about 0.58 eV, and this result is consistent with the value reported in our previously published article [28]. When R5011 was doped individually or jointly with S5011 into E44, the *E*_a_ increased with increasing R5011 concentration. In the E44/R5011 samples, the *E*_a_ value increased from 0.577 to 0.642 eV as the concentration of R5011 increased from 0 to 4.0 wt.%. Conversely, the activation energies of the E44/S5011 samples showed virtually the same value (0.585 ± 0.006 eV). Based on this result, we believe that the R5011 material we used as received contained a considerably higher content of impurity ions. The origin of the excess ions could be the source (viz. the manufacturer) from the very beginning. It is more likely to have originated from later contamination (say, moisture from the air) in the lab, or degradation over time due to unknown environmental conditions. Similar to ion diffusivity, the DC conductivity as a function of temperature exhibited the Arrhenian behavior as well (Figure 7c). It is clear from Figure 7c that at 30 °C *σ*_dc_ of the E44/R5011/S5011 samples were higher than those of pure E44 (14.9 × 10^−9^ S·m^−1^). With increasing binary-dopant concentration from 1.0 to 4.0 wt.%, the *σ*_dc_ values of the E44/R5011/S5011 samples grew from 26.6 × 10^−9^ to 76.6 × 10^−9^ S·m^−1^.

Additional examinations of excess or dissociated ions from chiral dopants in CLCs and their influences on device performance should be carefully explored in future studies. Although impurity ions in TFT-grade LC materials are absolutely undesired in active-matrix display devices, the benefits of a sufficient quantity of ions can be found in some other LC applications for particular purposes. The electrohydrodynamic effect induced by the flow of ions in response to an applied low-frequency electric field can be utilized to generate new optical states. For example, the oscillation of space charges or ions in a CLC material made from a nematic LC, E7, dispersed separately with R5011 and S5011, evoked several optical states including the focal conic, uniform lying helix, and dynamic scattering states, which were characterized optically, electrically, and thermally [43]. The flow of space charges enabled the alignment of the helix of a CLC composed of a nematic LC, 20 wt.% of a chiral dopant (S811), and chemical agents for polymerization stability, created a grid-like optical state that featured wider viewing angle for the reflected Bragg wavelengths [44]. A switchable smart window was created using electrohydrodynamic instability of space charges in a nematic LC doped with S811, allowing the electrical and thermal tuning by switching between Grandjean planar and dynamic scattering states [13].

### 3.4. Dielectric Properties of CLCs in Antiparallel and 90°-Twisted LC Cells

CLC material has been employed in antiparallel and 90°-twisted LC cell structures [45,46]. To investigate the role of the LC cell structure (namely, the cell mode) in the ionic behavior dominated by a chiral dopant, specific concentrations of R5011 and S5011 were selected to prompt right- and left-handed chiral nematic structures, respectively, in both the antiparallel (viz., 180°-twisted) and 90°-twisted cell modes.

**Figure 8:**
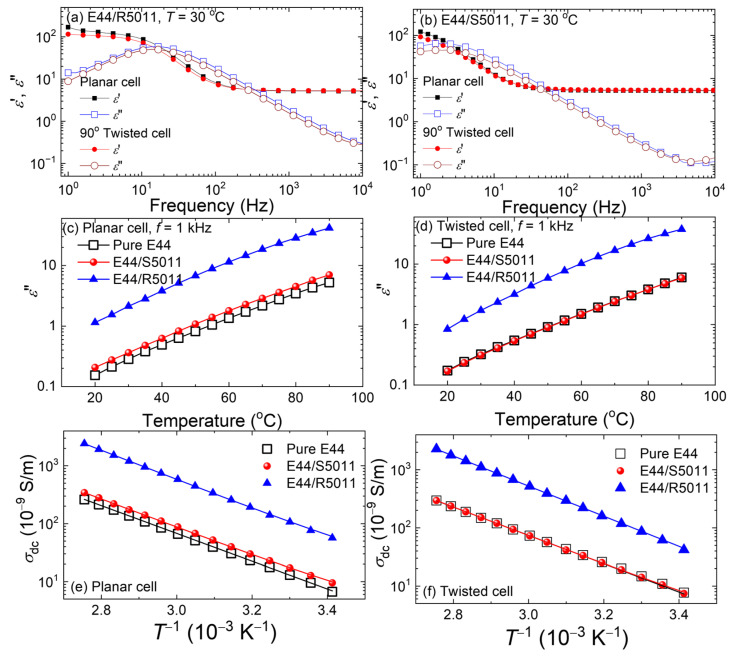
Dielectric spectra in the frequency range of 1 Hz–10 kHz of E44 doped with (**a**) 2.62 wt.% of R5011 and (**b**) 2.57 wt.% of S5011 in antiparallel and 90°-twisted LC cells at 30 °C, *T*-dependent *ε*″ at 1 kHz of the pure E44 and doped E44 in (**c**) the planar and (**d**) 90°-twisted cell modes at temperatures between 20 and 90 °C, and *σ*_dc_ in the natural logarithmic scale of the neat E44 and doped counterparts in (**e**) planar and (**f**) 90°-twisted LC cells against the inverse absolute temperature (in K^−1^). Straight solid lines in both (**e**,**f**) are the fitted curves following the Arrhenius equation.

Figure 8a,b present the real and imaginary parts of the dielectric spectra measured from E44 incorporated with 2.62 wt.% R5011 and 2.57 wt.% S5011, respectively, in the antiparallel and 90°-twisted cell modes at *T* = 30 °C. Both the *ε*′ and *ε*″ spectra of either CLC in antiparallel surface alignment were comparable to those in 90°-twisted surface alignment in the frequency range of 1–10^4^ Hz. For a typical LC cell, the sources of ions are mainly from the LC material, the alignment layers, and the electrode surfaces. Since our antiparallel and 90°-twisted LC cells are identical in terms of these material parameters, the cell-mode effect was inoperative in the dielectric properties of the left- or right-handed CLC. Figure 8c,d illustrate the *T*-dependent *ε*″ of the three samples—pristine E44 and E44 doped separately with 2.62 wt.% R5011 and S5011—in the antiparallel and 90°-twisted cell modes, respectively. Here the dielectric data were acquired at 1 kHz over a temperature range of 70 °C. The thermal response of *ε*″ for the three mesogenic materials did not show a significant disparity between the antiparallel and 90°-twisted cell structures. The distinct *ε*″ curve of the E44/R5011 sample was noticeable in comparison with that of the E44/S5011 sample, which showed a similar thermal change to that of pure E44. In accordance with Figure 8c, the enhanced ionic response by doping with R5011 was manifested by the promoted *ε*″ value of 1.14 from 0.15 (pure E44) at 20 °C, while S5011 produced a negligible increase of merely 0.02. Moreover, the *T*-dependent *ε*″ ranged increasingly from 1.14 to 41.7 for E44/R5011 and from 0.2 to 7 for E44/S5011 with escalated *T* from 20 to 90 °C. It is worth mentioning that the *ε”* values acquired from 90°-twisted cells were slightly smaller than those from antiparallel cells, with a maximal difference of 0.3 at 20 °C for all examined samples (Figure 8d). Considering the strong surface anchoring in both types of cells, the slight increase in the *ε*′ (*f*) and *ε*” (*f*) values of the antiparallel structure is ascribable to the trivial discrepancy between the cell gaps—6 μm of the antiparallel cell and 5 μm of the 90°-twisted cell. The obvious blueshift of the dielectric spectra (Figure 8a,b) could be mainly explained by the non-negligible ionic influence of R5011, whereas the chiral-dopant S5011 did play a conspicuous role in altering the dielectric spectrum of both structures as afore-discussed (Figure 4b).

The thermal increase in DC conductivity of pristine E44 and E44 doped with R5011 or S5011 can be elicited from Figure 8e,f, which complied well with the Arrhenius equation. In antiparallel LC cells, the DC conductivity in pure E44, *σ*_dc_ (E44) = 6.9 × 10^−9^ S·m^−1^, was of the same order of *σ*_dc_ (E44/S5011) = 9.9 × 10^−9^ S·m^−1^ and smaller than *σ*_dc_ (E44/R5011) = 6.0 × 10^−8^ S·m^−1^ by one order of magnitude at 20 °C. Nearly the same results were observed in the other cell mode (i.e., the case of 90°-twisted cells). Because of the dependence of DC conductivity on the ion density and mobility, the effect of temperature on the ion mobility is strongly denoted by the increase in *σ*_dc_, irrespective of the LC cell structure. The considerable ion density in doped E44 on account of R5011 raised DC conductivity compared with the samples of E44 or the counterpart doped with S5011.

### 3.5. Optical Characteristics of CLCs in Antiparallel and 90°-Twisted LC Cells

**Figure 9:**
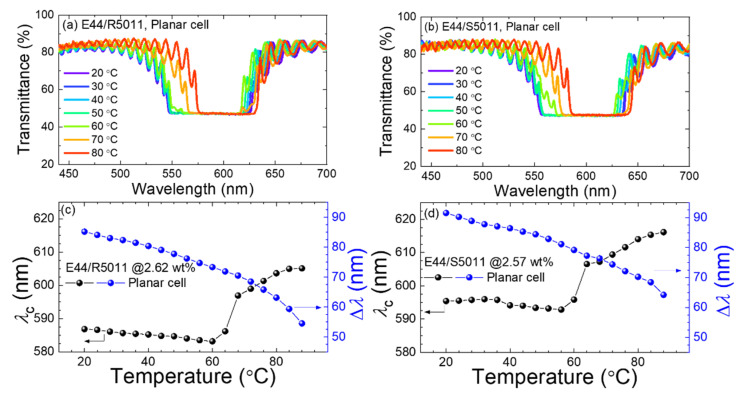
Transmission spectra of E44 doped with (**a**) 2.62-wt.% R5011 and (**b**) 2.57-wt.% S5011 and Bragg’s bandgap properties of (**c**) the right-handed and (**d**) left-handed CLCs at temperatures ranging from 20 to 90 °C.

Figure 9a,b highlights the optical transmission spectra of a right-handed CLC (consisting of E44 and 2.62-wt.% R5011) and a left-handed CLC (made of E44 doped with 2.57-wt.% S5011), respectively, sandwiched in antiparallel LC cells in a temperature range of 20–90 °C. The pitch lengths *p* of the two CLCs can be calculated by plugging the chiral dopant concentration *c* and HTP (= 107.5 μm^−1^) into the relationship

(7) $$p=\frac{1}{\left|\mathrm{HTP}\right|\cdot c},$$

resulting in *p* = 0.355 μm for E44/R5011 and *p* = 0.362 μm for E44/S5011. The spectrum from the planarly aligned E44/R5011 cell resembled that of the 90°-twisted sample at a given temperature, and the central wavelength of the Bragg reflection band, *λ*_c_ = 585 ± 1 nm, for both LC cell structures at 20 °C. For the E44/S5011 mixture in both the antiparallel and 90°-twisted cell modes, *λ*_c_ = 593 ± 3 nm at 20 °C. Evidently, the reflection-band characteristics of the right- and left-handed CLCs with a chiral dopant concentration of *c* ≈ 2.6 wt.% are not essentially affected by surface alignment of dissimilar cell structures. The large *d*/*p* ratio of 15 of the examined CLCs ensures a significant number of periodically helical turns for the incident light to traverse across the CLC interlayers, so that the spectrum can be intrinsically determined regardless of the cell configuration as a device parameter. To perceive how the spectral features varied with *T*, the central wavelength *λ*_c_ and bandgap width Δ*λ* are plotted in Figure 9c,d for E44/R5011 and E44/S5011, respectively. Figure 9c manifests the reduction in Δ*λ* from 85.6 to 54.7 nm with elevated *T* from 20 to 90 °C, which can be attributed to diminishing birefringence Δ*n* caused by an increasing degree of disordering (or randomness of molecular orientation) of the mesophase as *T* getting closer to the transition temperature *T*_c_. Similarly, the left-handed counterpart (E44/S5011) displayed a narrowing in width of the Bragg reflection band with rising *T* (Figure 9d). On the other hand, the observed central wavelength from E44/R5011 exhibited a stable response to *T* between 20 and 60 °C (Figure 9b), triggering a minimal blueshift from 586.2 nm at 20 °C to 582.6 nm at 60 °C (with the maximal difference in *λ*_c_ between the antiparallel and 90°-twisted LC cells to be smaller than 5 nm). In an earlier study, this thermal stability was tracked in a narrower *T* range of 20–40 °C for a nematic LC doped with R5011 [47]. Furthermore, to give the LC/R5011 mixture a wider wavelength range of thermal response or tunability, another chiral dopant known to possess a strongly *T*-dependent HTP was incorporated [48]. Here the marginal blueshift could be attributable to the limited decline in average refractive index 〈*n*〉 [49] according to *λ*_c_ = *p*〈*n*〉, where 〈*n*〉 ≡ (*n*_e_ + *n*_o_)/2, with increasing *T* for a substantially constant pitch length. At *T* > 60 °C, a redshift in *λ*_c_ occurred, increasing from 586.2 to 603 nm as *T* increased from 60 to 90 °C for the 90°-twisted cell. This phenomenon is not rarely encountered even though CLC materials typically possess the thermal property of d*p*/d*T* < 0, yielding a smaller *λ*_c_ value—namely, a shorter Bragg wavelength—at a higher temperature in the Grandjean planar texture. For E44/S5011, *λ*_c_ was quite stable from 20 to 56 °C for the antiparallel structure and to 64 °C for the 90°-twisted structure. The redshift in *λ*_c_ beyond 56–64 °C inferred the elongation of the helical pitch with increasing *T*, which was reported in a previous work involving S5011 as a chiral dopant [50]. In light of Figure 9c,d, a thorough investigation into the mechanisms responsible for the sharp rise in the *T*-dependent *λ*_c_ plots around 60 °C is highly desirable. Overall, unlike the dielectric results discussed in preceding subsections, small variance in the optical properties between R5011- and S5011-doped cells is easily understood by virtue of the minor difference in concentration (of barely 0.05 wt.%), which was difficult to be precisely controlled.

## 4. Conclusions

A thermal study of a nematic LC (E44) doped separately with enantiomeric chiral agents (R5011 and S5011) was conducted using dielectric spectroscopy and transmission spectrometry. According to the temperature-dependent dielectric data, the phase transition temperature (i.e., clearing point) decreased by up to 8 °C with increasing dopant concentration up to 4.0 wt.% for both right- or left-handed CLCs. A new aspect of investigation was highlighted by exploring the role of each chiral dopant in the ionic properties of the resulting CLC. It was found that the handedness of the chiral dopant was not directly associated with the detected ionic response corresponding to the concentration of the added chiral agent. The dielectric data and the related key parameters unraveled that our CLCs containing R5011 were much more ion-rich in comparison with the nematic host E44 and its left-handed counterparts at various dopant concentrations. A binary mixture of equal amounts of R5011 and S5011 were concocted with the host E44 to form a racemic blend. The ionic properties of the binarily doped sample were quite similar to the singly doped samples comprising R5011. Contributed evidently by R5011, the higher density of ions detected in the E44/R5011 mixtures may have originated from uncontrolled subsequent contamination or degradation of the dopant material over time. It was confirmed that the LC cell mode, either antiparallel or twisted by 90° for surface alignment, had no significant influence on the observed ionic behavior. On the basis of the temperature-dependent spectral data acquired from two opposite-handed CLCs, the elongation of helical pitch (i.e., weakening of HTP) was monitored at *T* above a certain temperature (*T* > ~60 °C). Unlike the distinguished dielectric properties of E44/R5011, the optical data of both E44/R5011 and E44/S5011 demonstrated virtually identical outcomes. Grounded in the presented experimental findings, we emphasize the potentially overlooked ionic contribution of a chiral additive in a constituted cholesteric liquid crystal.

## Figures and Tables

**Table 1:** Activation energies *E*_a_ of E44 impregnated with various concentrations of a single or binary chiral dopant.

Dopant Concentration (wt%)	*E*_a_ (eV)		
	E44/R5011	E44/S5011	E44/R5011/S5011
0	0.577	0.584	0.577
1	0.602	0.590	0.599
2	0.620	0.579	0.604
4	0.642	0.586	0.655

## Data Availability

The authors confirm that the data supporting the findings of this study are available within the article.
